# Effect of visual endotracheal tube combined with bronchial occluder on pulmonary ventilation and arterial blood gas in patients undergoing thoracic surgery

**DOI:** 10.3389/fsurg.2022.1040224

**Published:** 2023-01-06

**Authors:** Jing Xin, Xiu-juan Fan

**Affiliations:** Department of Anesthesiology, Ordos Center Hospital. Ordos, China

**Keywords:** thoracic surgery, visual endotracheal tube, bronchial occluder, pulmonary ventilation, arterial blood gas

## Abstract

**Background:**

To investigate the effect of visual endotracheal tube combined with bronchial occluder on pulmonary ventilation and arterial blood gas in patients undergoing thoracic surgery.

**Methods:**

Ninety patients who underwent thoracic surgery under anesthesia and required pulmonary ventilation at our hospital from May 2020 to December 2021 were collected. The patients were divided into three groups according to different intubation methods: visual double-lumen endotracheal tube group (VDLT group), bronchial occluder group (BO group), and VDLT + BO group. Clinical data and laboratory test data were collected from the three groups. Additionally, the three groups were compared in terms of peak airway pressure, time to correct positioning, pulmonary ventilation time, hemodynamics before and after intubation, intubation success rate, and postoperative recovery.

**Results:**

The VDLT + BO group was superior to the BO group or VDLT group in airway peak pressure, time to correct positioning, pulmonary ventilation time, intubation success rate, and hemodynamics after intubation (*P* < 0.05). In the comparison of postoperative recovery, the postoperative pain score, white blood cell level, incidence rate of pneumonia, hospital stay and hospitalization costs in the VDLT + BO group were significantly lower than those in the BO group or VDLT group (*P* < 0.05).

**Conclusion:**

The visual endotracheal tube combined with bronchial occluder is effective in pulmonary ventilation during thoracic surgery under anesthesia, and can improve arterial blood gas in patients.

## Introduction

At present, about 200,000 cases of thoracic surgery are performed in China each year, and the number is increasing at a rate of 5%–7% per year. Thoracoscopic surgery has many advantages such as small incision, wide surgical field, rapid postoperative recovery, and therefore has been widely applied in clinical practice (1). The key to thoracoscopic surgery is lung isolation and one-lung ventilation. Lung isolation is required to ensure ventilation and to prevent entry of blood, secretions, and tumor necrotic tissue from an affected lung into a non-diseased lung. Additionally, by using lung isolation techniques, fully lung collapse of the affected lung is achieved, contributing to improving surgical exposure and subsequent surgical procedures.

There are two common methods of lung isolation. The first is a double-lumen tube (DLT). It can effectively isolate both lungs to provide independent ventilation for each lung, can provide a sucker for sputum in the isolated lung, and effectively prevent various secretions from flowing to the healthy lung, especially suitable for patients with wet lung and empyema (2). However, due to the large outer diameter of the tube, intubation of DLT is relatively difficult and results in many problems and complications, such as hypoxemia, increased peak airway pressure, and airway injury (3). The second is a bronchial occluder (BO). BO is inserted into one bronchus (left or right) through an endotracheal tube, and then the cuff is inflated to seal the bronchus and achieve lung isolation (4). BO is more and more widely used because of its advantages of easy intubation, easy positioning, little injury and satisfactory occlusion effect, acting as an alternative to DLT (5, 6).

An endotracheal tube mounted camera is a new intubation tool with an embedded high-definition camera in the anterior wall at the tip of tube, along with a monitor connected to the camera, LED light, and a unique flush port for cleaning the camera (7). In patients without a difficult airway, the use of a visual endotracheal tube allows safe and rapid endotracheal intubation while not requiring laryngoscopic assistance (8, 9). However, there are no Chinese reports on the effect of visual endotracheal tube combined with BO on lung ventilation in thoracic surgery under anesthesia and its effect on arterial blood gas in patients. Therefore, this study comprehensively analyzed the clinical safety and advantages of the combination by measuring peak airway pressure, time to correct positioning, pulmonary ventilation time, hemodynamics and postoperative recovery.

## Materials and methods

### General information

We collected 90 patients undergoing thoracic surgery under anesthesia at our hospital and requiring pulmonary ventilation from May 2020 to December 2021 for a prospective randomized controlled trial. According to the random number table, they were divided into three groups: VDLT group (*n* = 30, visual DLT), BO group (*n* = 30, BO), VDLT + BO group (*n* = 30, visual DLT + BO).

Inclusion criteria were: (1) Patients who need thoracic surgery; (2) Aged 18–80 years old; (3) Normal pulmonary function or mild ventilation dysfunction; (4) The American Society of Anesthesiologists (ASA) class I-IV. Exclusion criteria were: (1) Patients with thoracic deformity or trauma; (2) Patients with abnormal cardiopulmonary function; (3) Bronchi variation with anatomical abnormality; (4) Suspected difficult airway, such as thyromental distance <6 cm, mouth opening <3 fingers, and Mallampati airway class III–IV; (5) Patients with malignant arrhythmia; (5) Patients with severe liver and kidney dysfunction; (6) Patients with mental illness, severe cognitive impairment or language problems. This study was approved by the ethics committee of our hospital, and both patients and their families understood the purpose of this study and had signed an informed consent. This study was approved by the Ethics Committee of Ordos Center Hospital (20200116).

### Anesthesia methods

All patients were admitted to the operating room for routine opening of venous access, followed by an infusion of lactated Ringer's balanced solution. Patients' heart rate (HR), non-invasive blood pressure (NIBP), oxygen saturation (SpO2), electrocardiogram (ECG), partial pressure of end-tidal carbon dioxide (PETCO2), and depth of anesthesia by Narcotrend. The endotracheal tube suitable for the patients was selected based on their gender, height, body mass, and surgical approach. Induction of anesthesia was achieved with intravenous injection of 0.05–0.07 mg/kg midazolam, 4 *μ*g/kg fentany l, 2 mg/kg propofol, and 0.2 mg/kg cisatracurium, and effective manual ventilation with a mask was given for 3 min. With completely relaxed muscle, the Narcotrend index of 37–64, and SpO2 reaching 100%, endotracheal intubation was started. All procedures were performed in cooperation with two experienced anesthesiologists.

### Method of intubation

In the VDLT group, the tube was inserted until it entered one of the main bronchus, and the cuff was inflated. The edges of the blue cuff, primary carina, and the other main bronchus were visible at the same time, suggesting correct placement. Additionally, the auscultation method proved successful tube positioning. Finally, the tube was fixed. In the BO group, the occluder was placed directly in the main bronchus requiring occlusion under the guidance of a fiberoptic bronchoscope. In the VDLT + BO group, the occluder was directly advanced to the target mainstem bronchus through the DLT mounted camera; both lungs were auscultated and the occluder position was continuously monitored. In the latter two groups, after confirming the correct position of the BO, one-lung ventilation was performed to adjust the respiratory parameters and the anesthesia ventilator was connected for volume-controlled ventilation. The position of the tube and occluder was monitored throughout the operation and adjusted in time if the surgical position changed.

### Outcome measures

The peak airway pressure, time to correct positioning and pulmonary ventilation time before and after intubation were compared among the three groups. Hemodynamic changes, including mean arterial pressure (MAP), HR, SpO2, arterial partial pressure of carbon dioxide (PaCO2), partial pressure of oxygen (PaO2), forced expiratory volume in 1 s (FEV1), and forced vital capacity (FVC), were compared. In addition, the airway class, and success rate of intubation as well as the pain score (10), white blood cell level, incidence rate of pneumonia, length of hospital stay and hospitalization costs of patients after thoracic surgery were compared among the three groups.

### Statistical analysis

SPSS 22.0 statistical software was used for statistical analysis. Measurement data were expressed as mean ± standard deviation (SD); A Student's t test was used for comparison between the two groups, and a one-way analysis of variance was used for comparison between multiple groups. Enumeration data were expressed as rates, and chi-square test was used for analysis. *P* < 0.05 was considered as statistically significant.

## Results

### Baseline characteristics of patients

A total of 90 patients receiving thoracic surgery were collected, divided into VDLT group (17 males, 13 females), BO group (19 males, 11 females), and VDLT + BO group (19 males, 11 females), with 30 cases in each group. There were no significant differences among the groups in age, gender, body mass index, ASA classification, systolic blood pressure, diastolic blood pressure, duration of hypertension, history of previous myocardial infarction, smoking history, history of alcoholism, complications, disease type, and preoperative levels of MAP, FEV1, and FVC (all *P* > 0.05), indicating that the groups were comparable (Table 1).

**Table 1:** Comparison of baseline characteristics of included patients.

Variables	VDLT group (*n* = 30)	BO group (*n* = 30)	VDLT + BO group (*n* = 30)
Age (year)	44.03 ± 9.39	45.80 ± 9.79	45.37 ± 11.70
Gender (male/female)	17/13	19/11	19/11
BMI (kg/m^2^)	23.19 ± 1.43	23.56 ± 0.95	23.39 ± 1.49
ASA classification			
I	0	0	0
II	10	8	11
III	17	20	16
IV	3	2	3
SBP (mmHg)	93.27 ± 7.29	92.33 ± 9.50	91.37 ± 10.16
DBP (mmHg)	148.60 ± 10.52	149.43 ± 12.76	148.57 ± 9.66
Course of hypertension (year)	3.47 ± 1.55	3.57 ± 1.92	3.60 ± 1.45
Previous myocardial infarction	4	6	5
History of smoking	17	15	14
History of alcoholism	14	12	12
With complications	10	9	12
Disease types			
Lung diseases	13	16	17
Bronchial diseases	13	10	9
Others	4	4	4
MAP (mmHg)	81.43 ± 6.05	81.65 ± 3.50	81.59 ± 3.69
HR (beats/min)	81.37 ± 7.96	81.10 ± 3.24	82.50 ± 3.39
SpO2 (%)	88.30 ± 4.50	87.95 ± 2.42	87.22 ± 2.40
PaCO2 (mmHg)	33.77 ± 6.85	32.07 ± 2.52	33.16 ± 1.62
PaO2 (mmHg)	73.73 ± 6.50	74.37 ± 4.18	73.54 ± 3.07
FEV1 (L)	2.38 ± 0.35	2.35 ± 0.41	2.37 ± 0.25
FVC (L)	3.27 ± 0.25	3.25 ± 0.27	3.23 ± 0.12

Values are mean ± SD or *n*. VDLT, visual double-lumen tube; BO, bronchial occluder; BMI, body mass index; ASA, American Society of Anesthesiologists; SBP, systolic blood pressure; DBP, diastole blood pressure; MAP, mean arterial pressure; HR, heart rate; SpO2, oxygen saturation; PaCO2, arterial partial pressure of carbon dioxide; PaO2, partial pressure of oxygen; FEV1, forced expiratory volume in 1 s; FVC, forced vital capacity.

### Comparison of peak airway pressure, time to correct positioning and pulmonary ventilation time

According to the comparison shown in Table 2, the peak airway pressure of patients in the VDLT + BO group was the smallest after intubation (22.64 ± 1.54 mmHg), significantly lower than that in the VDLT group (27.08 ± 1.51 mmHg) and the BO group (24.70 ± 1.70 mmHg) (*P* < 0.01). Also, the VDLT + BO group had the shortest time to correct positioning and longest pulmonary ventilation time (*P *< 0.01).

**Table 2:** Comparison of peak airway pressure, time to correct positioning and pulmonary ventilation time before and after intubation .

Variables		VDLT group (*n* = 30)	BO group (*n* = 30)	VDLT + BO group (*n* = 30)	Statistics	*P*
Peak airway pressure (mmHg) time to correct positioning (s)	T1	20.49 ± 1.24	20.98 ± 1.38	20.26 ± 1.45	2.192	0.118
	T2	27.08 ± 1.51^a^	24.70 ± 1.70^a^	22.64 ± 1.54	59.123	0.000
		6.97 ± 1.27^a^	5.20 ± 0.71^a^	4.07 ± 1.11	57.099	0.000
Pulmonary ventilation time (min)		117.40 ± 14.79^a^	138.43 ± 14.83^a^	153.17 ± 14.09	45.658	0.000

Values are mean ± SD.

^a^ *P* < 0.01 *vs*. VDLT + BO group. VDLT, visual double-lumen tube; BO, bronchial occluder; T1, before intubation; T2, after intubation.

### Comparison of the success rate of intubation for different airway classes

As shown in Table 3, in patients with different airway classes, the VDLT + BO group had the highest number of patients who completed intubation (27/30), but there was no significant difference among the groups (*P* > 0.05).

**Table 3:** Comparison of the success rate of intubation for different airway classes.

Variables		VDLT group (*n* = 30)	BO group (*n* = 30)	VDLT + BO group (*n* = 30)
Airway class	I	66.7 (10/15)	75.0 (3/4)	100.0 (1/1)
	II	80.0 (12/15)	84.6 (22/26)	89.7 (26/29)
Success rate of intubation (%)		73.3 (22/30)	83.3 (25/30)	90.0 (27/30)

Values are percentage (*n* / total *n*). VDLT, visual double-lumen tube; BO, bronchial occluder.

### Comparison of hemodynamic parameters

As shown in Table 4, there was no significant difference in all hemodynamic parameters before intubation among the three groups (*P* > 0.05). After tracheal intubation, MAP, HR, SpO2, PaCO2, PaO2, FEV1 and FVC in the VDLT + BO group were significantly better than those in the BO group and the VDLT group, with the worst performance in the VDLT group (*P* < 0.05).

**Table 4:** Comparison of hemodynamic parameters before and after intubation.

Variables	VDLT group (*n* = 30)	BO group (*n* = 30)	VDLT + BO group (*n* = 30)	Statistics	*P*
MAP (mmHg)					
T1	81.40 ± 6.12	81.44 ± 3.40	81.29 ± 3.75	0.009	0.991
T2	88.06 ± 5.76^a^	86.74 ± 3.87*	84.08 ± 3.55	6.047	0.003
HR (beats/min)					
T1	81.37 ± 8.00	81.20 ± 3.09	82.53 ± 3.33	0.561	0.573
T2	89.33 ± 7.58^a^	85.73 ± 3.37	84.27 ± 3.38	7.626	0.001
SpO2 (%)					
T1	88.57 ± 4.60	87.89 ± 2.44	87.06 ± 2.38	1.575	0.213
T2	93.82 ± 4.22^a^	91.15 ± 2.55*	89.07 ± 2.35	17.106	0.000
PaCO2 (mmHg)					
T1	33.87 ± 6.72	31.87 ± 2.37	32.96 ± 1.83	1.661	0.196
T2	37.85 ± 6.81^a^	40.11 ± 2.59	41.84 ± 2.00	6.317	0.003
PaO2 (mmHg)					
T1	73.90 ± 6.71	74.54 ± 4.40	73.50 ± 3.03	0.332	0.719
T2	77.18 ± 6.71^a^	81.03 ± 4.33	83.36 ± 3.13	11.910	0.000
FEV1 (L)					
T1	2.39 ± 0.35	2.34 ± 0.43	2.38 ± 0.26	0.152	0.859
T2	2.71 ± 0.35^a^	3.11 ± 0.38^a^	3.36 ± 0.25	28.902	0.000
FVC (L)					
T1	3.27 ± 0.27	3.24 ± 0.28	3.22 ± 0.16	0.317	0.729
T2	3.50 ± 0.27^a^	3.83 ± 0.29^a^	4.13 ± 0.13	50.452	0.000

Values are mean ± SD.

*P < 0.05 vs. VDLT + BO group.

^a^ *P* < 0.01 *vs*. VDLT + BO group. VDLT, visual double-lumen tube; BO, bronchial occluder; MAP, mean arterial pressure; HR, heart rate; SpO2, oxygen saturation; PaCO2, arterial partial pressure of carbon dioxide; PaO2, partial pressure of oxygen; FEV1, forced expiratory volume in 1 s; FVC, forced vital capacity; T1, before intubation; T2, after intubation.

### Comparison of postoperative recovery

Finally, we compared the postoperative recovery of patients in the three groups (Table 5). Patients in the VDLT + BO group had significantly lower postoperative pain scores, lowest incidence of infection, longer hospital stays, and required less costs than patients compared with the VDLT group and the BO group.

**Table 5:** Comparison of postoperative recovery.

Variables	VDLT group (*n* = 30)	BO group (*n* = 30)	VDLT + BO group (*n* = 30)	Statistics	*P*
Pain score	5.30 ± 0.65	4.63 ± 0.72	3.83 ± 1.02	24.506	0.000
Abnormal rate of white blood cells (%)	20 (66.7)	14 (46.7)	7 (23.3)	11.379	0.000
Incidence of pneumonia (%)	9 (30.0)	4 (13.8)	2 (6.7)	6.115	0.047
Length of hospital stay (day)	7.50 ± 1.01	5.03 ± 0.76	4.33 ± 1.15	84.831	0.000
Hospitalization costs (Chinese Yuan)	9224.92 ± 1534.93	7392.93 ± 973.10	6661.40 ± 1517.37	27.999	0.000

Values are mean ± SD or *n* (%). VDLT, visual double-lumen tube; BO, bronchial occluder.

## Discussion

DLT has the advantages of good lung isolation, safety, and easy fixation, but it has a large diameter, special shape, and hard texture. DLT is difficult to operate, easily causing greater stimulation to the body during intubation and extubation, damage to the airway, increased airway pressure, and prolonged time to correct positioning (11–13). Therefore, DLT is not conducive to stable progress of thoracic surgery. As a new lung isolation method and new one-lung ventilation technique, BO is selected by clinicians and patients because of its small outer diameter and 50% increase in the area of one-lung ventilation tube compared with DLT, which just compensates for the disadvantages of DLT (2, 14). In this study, a combination of visual DLT and BO was innovatively used for lung isolation. Such a combination can obtain clear real-time visual monitoring during intubation and the advantages of both methods.

At present, the application advantages of visual endotracheal tube combined with BO in clinical surgery are significant. Liang (15) et al. compared the visual single-lumen endotracheal tube combined with BO in thoracic surgery with common single-lumen endotracheal tube combined with BO, and found that the former was superior in intraoperative time to correct positioning, postoperative pneumonia infection rate and postoperative airway mucosal injury degree. In our study, patients who received a combination of visual DLT and BO had significantly better rates of postoperative pulmonary infection, less postoperative pain, shorter length of hospital stay, and lower costs than those who received visual DLT or BO alone.

Peak airway pressure refers to the highest airway pressure in mechanical ventilation, and once BO cuff displaces, peak airway pressure is directly affected, which has also been a problem for clinicians (16, 17). Our study found that patients who underwent thoracic surgery with visual DLT combined with BO intubation had the smallest peak airway pressure after surgery (22.64 ± 1.54 mmHg), while patients who were intubated with visual DLT alone had the largest value. Additionally, the ADLT + BO group was superior to the other two groups in time to correct positioning, pulmonary ventilation time, airway classification and intubation success rate as well as hemodynamic comparison, and the performance of BO alone was better than visual DLT alone. Similarly, Xu (18) et al. found that the time to correct positioning and intubation time of BO alone were shorter than those of common DLT, and the airway pressure was lower. However, a meta-analysis (19) showed that compared with common DLT alone in thoracic surgery, BO alone required shorter time to correct positioning and was safer, but was associated with higher incidence and severity of airway injury. Anesthesiologists' familiarity with airway anatomy and proficiency in the procedure brings some difference in effect.

Collectively, our study demonstrates that the visual DLT combined with BO provides good ventilation, better hemodynamics, and high safety. Therefore, it shows high application value as an important method to achieve lung isolation; endobronchial blockers suitable for specific surgery procedure should be selected based on their characteristics and indications, thus minimizing the damage to patients.

### Limitation

The limitation of this study is the lack of observation of long-term prognosis of patients, and the types of thoracic surgery performed inside the groups were different, which existed certain bias when exploring outcomes such as postoperative complications, postoperative length of stay, or post-extubation values of pulmonary performance and gas exchange. These will be further improved in future studies, requiring large multicenter cohorts and more in-depth studies using prospective settings.

## Conclusion

In summary, during thoracic surgery under anesthesia, visual endotracheal tube combined with BO offers the advantages of lower peak airway pressure and better performance in oxygenation, hemodynamic parameters, postoperative recovery of patients. Therefore, such a combination is a good choice to safely and effectively achieve single-lung ventilation during thoracic surgery under anesthesia.

## Data Availability

The original contributions presented in the study are included in the article/Supplementary Material, further inquiries can be directed to the corresponding author/s.
